# Emergence and Characterization of a Novel IncP-6 Plasmid Harboring *bla*_KPC–2_ and *qnrS2* Genes in *Aeromonas taiwanensis* Isolates

**DOI:** 10.3389/fmicb.2019.02132

**Published:** 2019-09-12

**Authors:** Xinjun Hu, Xiao Yu, Yibing Shang, Hao Xu, Lihua Guo, Yile Liang, Yixin Kang, Li Song, Jifeng Sun, Feng Yue, Yimin Mao, Beiwen Zheng

**Affiliations:** ^1^Department of Infectious Diseases, The First Affiliated Hospital, College of Clinical Medicine, Henan University of Science and Technology, Luoyang, China; ^2^Collaborative Innovation Center for Diagnosis and Treatment of Infectious Diseases, State Key Laboratory for Diagnosis and Treatment of Infectious Diseases, The First Affiliated Hospital, College of Medicine, Zhejiang University, Hangzhou, China; ^3^Department of Respiratory Medicine, The First Affiliated Hospital, College of Clinical Medicine, Henan University of Science and Technology, Luoyang, China

**Keywords:** *Aeromonas taiwanensis*, *bla*_KPC–2_, *qnrS2*, whole-genome sequencing, plasmid analysis

## Abstract

The dissemination of *Klebsiella pneumoniae* carbapenemases (KPCs) among Gram-negative bacteria is an important threat to global health. However, KPC-producing bacteria from environmental samples are rarely reported. This study aimed to elucidate the underlying resistance mechanisms of three carbapenem-resistant *Aeromonas taiwanensis* isolates recovered from river sediment samples. Pulsed-field gel electrophoresis (PFGE) and whole genome sequencing (WGS) analysis indicated a close evolutionary relationship among *A. taiwanensis* isolates. S1-PFGE, Southern blot and conjugation assays confirmed the presence of *bla*_KPC–__2_ and *qnrS2* genes on a non-conjugative plasmid in these isolates. Plasmid analysis further showed that pKPC-1713 is an IncP-6 plasmid with a length of 53,205 bp, which can be transformed into DH5α strain and mediated carbapenems and quinolones resistance. The plasmid backbone of p1713-KPC demonstrated 99% sequence identity to that of IncP-6-type plasmid pKPC-cd17 from *Aeromonas* spp. and IncP-6-type plasmid: 1 from *Citrobacter freundii* at 74% coverage. A 14,808 bp insertion sequence was observed between *merT* gene and hypothetical protein in p1713-KPC, which include the quinolone resistance *qnrS2* gene. Emergence of plasmid-borned *bla*_KPC_ and *qnrS2* genes from *A. taiwanensis* isolates highlights their possible dissemination into the environment. Therefore, potential detection of such plasmids from clinical isolates should be closely monitored.

## Introduction

The global spread of *Klebsiella pneumoniae* carbapenemases (KPCs) among Gram-negative bacteria, has become a major public health concern in recent decades (Grundmann et al., 2017). Infections caused by KPC-producing bacteria have been associated with a high mortality rate of 53%, which presents tremendous challenges for clinicians and healthcare providers (Munoz-Price et al., 2013). Although most KPCs are commonly found in *Enterobacteriaceae*, the production of KPCs appeared to be less dominance in *Aeromonadaceae* spp. (Picao et al., 2013; Montezzi et al., 2015; Lamba and Ahammad, 2017; Paschoal et al., 2017; Tuo et al., 2018; Xu et al., 2018; Zheng et al., 2018b). The emergence of *bla*_KPC_ gene on broad-host-range plasmids has facilitated its rapid dissemination to *Enterobacteriaceae* and other Gram-negative families (van Duin and Doi, 2017). More importantly, KPC-type carbapenemases are often associated with quinolone resistance determinants, which can be modulated by *qnrS* gene (Montezzi et al., 2015). The quinolone resistance gene, *qnrS2*, shares 92% identity with the *qnrS* gene, was first detected on a highly mobile plasmid that is isolated from wastewater treatment plant bacterial population (Bonemann et al., 2006). Thus far, the co-existence of *bla*_KPC_ and *qnrS2* in the same plasmid has not been reported.

The objective of the present study was to identify plasmid-borne *bla*_KPC–__2_ genes in *Aeromonas taiwanensis* from river sediments in China and to analyze the plasmids carrying them. Three strains were isolated and found to harbor IncP-6 plasmids carrying both *bla*_KPC–__2_ and *qnrS2*. We characterized the phenotypic and molecular features of these strains in order to assess their genomic epidemiology profiles. Additionally, the complete nucleotide sequence of p1713-KPC was determined by plasmid sequencing. To the best of our knowledge, this is the first study to indicate the co-occurrence of *bla*_KPC–__2_ and *qnrS2* genes in the same plasmid and this plasmid harboring these two gene were founded in *A. taiwanensis* isolates for the first time.

## Materials and Methods

### Bacterial Isolation and Pulsed-Field Gel Electrophoresis (PFGE)

The KPC-2-producing *A. taiwanensis* isolates were previously identified from river sediment samples from China (Xu et al., 2018). A total of three *A. taiwanensis* isolates were further characterized to uncover the underlying resistance mechanisms. The genomic diversity of *A. taiwanensis* isolates was assessed by *Xba*I-pulsed-field gel electrophoresis (PFGE) as described previously (Zheng et al., 2016). A dendrogram of PFGE profiles was constructed with BioNumerics v7.6 by using UPGMA (unweighted pair group method with averages) clustering. Isolates with a similarity cut-off of ≥80% were considered as pulsotypes.

### Whole-Genome Sequencing (WGS) and Assembly

To characterize the genomic features and resistome of *A. taiwanensis* isolates, WGS was conducted on all the three isolates. Genomic DNA was extracted and sequenced with Hiseq X Ten sequencing platform (Illumina, San Diego, CA, United States). Subsequently, *de novo* genome assembly and bioinformatics analysis were carried out as previously described (Xu et al., 2018; Zheng et al., 2018a). After obtaining the raw reads, the genome sequence of pKPC-1713 was assembled using plasmidSPAdes (Antipov et al., 2016).

### Phylogenetic Reconstruction and Analysis

The pan-genome analysis was performed with Roary: the pangenome pipeline (version 3.6.0) using the Prokka annotation (Seemann, 2014; Page et al., 2015). The complete genome sequences of all 20 *Aeromonas* spp. strains were downloaded from NCBI genome database (current as of March 30, 2019). Roary software was used to cluster the above genomes, The software clustered the genomes based on the genes carried by each strain. According to the distribution of each gene among the strains, the genes were divided into core genes and accessory genes. Core genes were defined as genes carried by 95% or more of the strains. Accessory genes were those carried by less than 95% of the strains. Then the core genome of these strains is obtained. A whole-genome phylogenetic tree was built from the core-genome SNPs of *Aeromonas* spp. and the three studied isolates. Phylogenetic reconstruction and analysis was performed using the R package phangorn (Schliep, 2011).

### Plasmid Characterization

The size of plasmid was determined by S1-nuclease PFGE analysis, as previously described (Zheng et al., 2015). Southern blot hybridization of plasmid DNA was performed on *Aeromonas hydrophila* isolates by using DIG-labeled *bla*_KPC_- and *qnrS*- specific probes, according to manufacturer’s instructions (Roche Diagnostics, Germany) (Zheng et al., 2016). Plasmid conjugation experiments were carried out by filter mating using *Escherichia coli* J53 and EC600 as recipient strains, at a ratio of 1:1 in broth culture (Zheng et al., 2015). The pKPC-1713 plasmid was transferred into chemically competent *E. coli* DH5α cells via transformation process. The transformants were selected on Luria-Bertani agar plates supplemented with 2 mg/L meropenem. The presence of *bla*_KPC_ and *qnrS2* genes was then screened by PCR and sequencing. Plasmid DNA was extracted from conjugants with Qiagen Plasmid Midi Kit (Qiagen, Valencia, CA). To obtain the complete sequence of the plasmid co-expressing *bla*_KPC_ and *qnrS2*, the combined application of PCR walking method with targeted primers (Supplementary Table S1) and Illumina sequencing technique were performed to specifically investigate the representative plasmid pKPC-1713. The RAST annotation pipeline was chosen to perform rapid annotation of the plasmids (Overbeek et al., 2014). Plasmid replicons and antibiotic resistance genes were identified using CGE server^1^. The sequence of pKPC-1713 was BLAST for homology against the NCBI plasmids database.

### Susceptibility Testing

The isolated *A. taiwanensis* 1713, *E. coli* DH5α and DH5α: pKPC-1713 were cultured overnight, while *E. coli* ATCC 25922 was used as a quality control. Minimum inhibitory concentrations (MICs) of amikacin, ampicillin, ampicillin-sulbactam, aztreonam, cefazolin, ceftazidime, cefotetan, ceftriaxone, ciprofloxacin, ertapenem, gentamicin, imipenem, levofloxacin, nitrofurantoin, piperacillin-tazobactam, tobramycin, and trimethoprim antibiotics were determined by VITEK 2 system with AST-GN16 panel. The results of antimicrobial susceptibility testing were interpreted according to 2017’s CLSI guidelines.

### Plasmid Mobilization

Plasmid conjugation was performed as previously described with some modification (Yi et al., 2012). *A. taiwanensis* isolates were electrotransformed with a plasmid pEC1002-MCR(CP021205) which contains a *tra* module. Isolates containing the resident and pEC1002-MCR plasmids were used as donor strains. Plasmid conjugative transfer was performed by using donor and recipient sodium azide resistant *E. coli* J53 cells mixed in a 1:1 ratio as described previously. Transconjugants were selected on MacConkey agar containing 2 mg/L imipenem and 100 mg/L sodium azide. Transconjugants were confirmed by susceptibility testing and PCR.

### Accession Numbers

The whole genome shotgun project of the *A. taiwanensis* isolates has been deposited into DDBJ/EMBL/GenBank under the Bioproject accession number PRJNA478520. The nucleotide sequence of plasmid p1713-KPC has been deposited into the GenBank database under GenBank accession number MH624132.

## Results and Discussion

### Comparative Genomic Analysis of Carbapenem-Resistant *A. taiwanensis* Isolates

**FIGURE 1:**
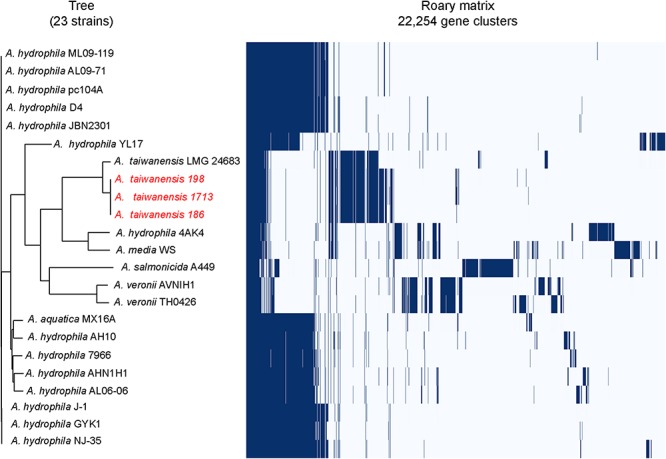
Genetic relatedness analysis of KPC-2-producing *Aeromonas* spp. isolates. Pan-genome analysis of *A. taiwanensis* isolates and other *Aeromonas* isolates by using Roary. The blue bars indicate the pan-genome of *Aeromonas* isolates, with 26,778 annotated genes detected in all the genomes analyzed. All the complete genome sequences of representative *Aeromonas* strains were downloaded from NCBI Genome database and used for phylogenetic analysis: *Aeromonas aquatica* strain MX16A (CP018201), *A. hydrophila* 4AK4 (CP006579), *A. hydrophila* strain AH10 (CP011100), *A. hydrophila* strain AHNIH1 (CP016380), *A. hydrophila* strain AL06-06 (CP010947), *A. hydrophila* AL09-71 (CP007566), *A. hydrophila* subsp. hydrophila ATCC 7966 (NC_008570), *A. hydrophila* strain D4 (CP013965), *A. hydrophila* strain GYK1 (CP016392), *A. hydrophila* J-1 (CP006883), *A. hydrophila* strain JBN2301 (CP013178), *A. hydrophila* ML09-119 (NC_021290), *A. hydrophila* NJ-35 (CP006870), *A. hydrophila* pc104A (CP007576), *A. hydrophila* YL17 (CP007518), *Aeromonas media* WS (CP007567), *Aeromonas salmonicida* subsp. salmonicida A449 (NC_009348), *Aeromonas taiwanensis* LMG 24683 (GCA_000820165), *Aeromonas veronii* strain AVNIH1 (CP014774), and *Aeromonas veronii* strain TH0426 (CP012504). *A. taiwanensis* isolates described in this study were colored as red.

Pulsed-field gel electrophoresis analysis revealed that two pulsotypes from *A. taiwanensis*, namely 198 and 186, exhibited relatively identical PFGE patterns (Supplementary Figure S1). Meanwhile, Roary matrix-based gene sequence analysis generated a large pan-genome matrix of 26,778 gene clusters across 23 genomes (Figure 1). Moreover, the 186, 198, and 1713 isolates were found to be genetically closely related by Roary matrix-based gene sequence analysis (Figure 1). These findings are consistent with the results of PFGE profiles, suggesting a possible clonal spread of KPC-producing *A. taiwanensis*. So far, only few reports described the detection of plasmid-mediated *bla*_KPC_ determinants in *Aeromonadaceae* (Picao et al., 2013; Montezzi et al., 2015). Notably, the presence of *bla*_KPC_ in *Aeromonadaceae* considered predominantly environmental is remarkable, and the spread of KPC-producing *A. taiwanensis* in aquatic environments deserves attention.

### Identification of the Plasmid Harboring Both *bla*_KPC–__2_ and *qnrS2* Genes

**FIGURE 2:**
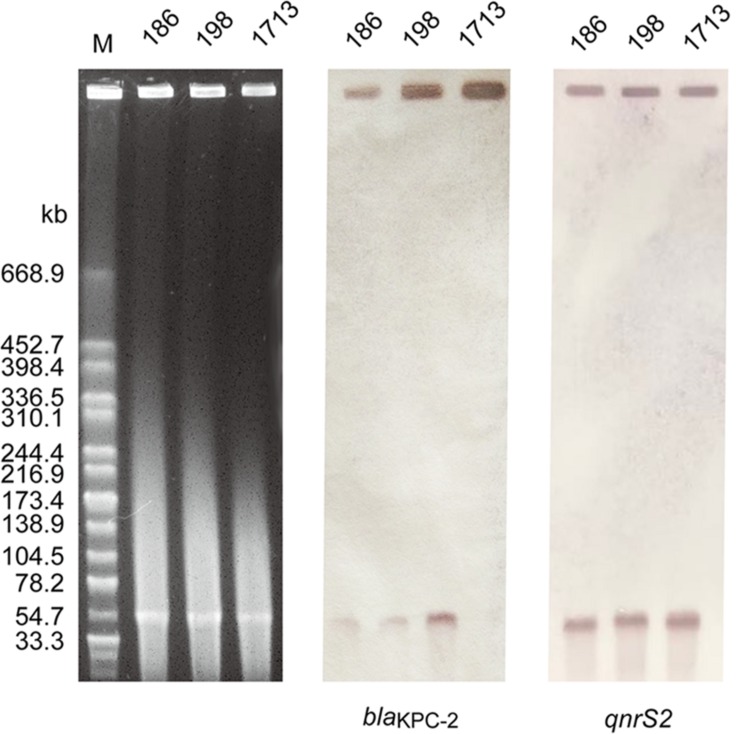
Plasmid profile and Southern blot hybridization of the three *A. taiwanensis* isolates. The southern blot hybridization of blaKPC and qnrS2 probes using S1 nuclease-digested DNA. Lane M: *Xba*I-digested genomic DNA fragments of *Salmonella enterica* serotype Braenderup H9812 as the size marker.

S1-PFGE and Southern blot analysis demonstrated that all *A. taiwanensis* isolates contained a ∼54 kb plasmid, harboring both *bla*_KPC–__2_ and *qnrS2* genes (Figure 2). Moreover, nearly identical plasmid sequences of the three isolates were assembled from WGS data by SPAdes (data not shown), suggesting that these isolates shared the same plasmid profile. Notably, none of the plasmids could be transferred to recipient strains via conjugation, despite repeated attempts, which suggests that *bla*_KPC–__2_ and *qnrS2* are located on a non-conjugative plasmid. Occasionally, *qnrS2* has been identified in the plasmids of *Aeromonas* spp. (Arias et al., 2010; Marti and Balcazar, 2012; Marti et al., 2014; Yang et al., 2017); however, the co-occurrence of *bla*_KPC_ and *qnrS2* genes in the same plasmid has never been described before. To our knowledge, this is the first study to indicate the co-occurrence of *bla*_KPC_ and *qnrS2* genes in the same plasmid. As a consequence, we selected a representative sample of 1713 isolate for further plasmid characterization.

Plasmid transformation and MICs determination revealed that carbapenem and quinolone resistant genes were successfully transferred from a donor strain to *E. coli* DH5α. Antibiotic susceptibility testing showed that the MIC values of ertapenem, imipenem, ciprofloxacin, and levofloxacin were increased from 0.125 to 8 mg/L, 0.125 to 16 mg/L, 0.25 to 8 mg/L, and 1 to 4 mg/L, respectively (Table 1). These results confirmed that carbapenem and quinolone resistance were successfully transferred to receipt cells.

**TABLE 1:** Characteristics of *A. taiwanensis* 1713 and its transformant strain^a^.

**Antibiotics**	***A. taiwanensis* 1713**	**DH5α: pKPC-1713**	***E. coli* E DH5α**
Ampicillin	≥128/R	≥128/R	1/S
Amoxillin/clavulanate	≥128/R	64/R	1/S
Piperacillin/tazobactam	≥128/R	≥128/R	1/S
Cefazolin	≥128/R	≥128/R	1/S
Ceftriaxone	≥128/R	≥128/R	0.125/S
Ceftazidime	16/R	16/R	0.25/S
Ceftaxitin	≥128/R	≥128/R	0.25/S
Aztreonam	≥128/R	≥128/R	0.25/S
Ertapenem	8/R	8/R	0.125/S
Imipenem	16/R	16/R	0.125/S
Ciprofloxacin	8/R	8/R	0.25/S
Levofloxacin	4/I	4/I	1/S
Amikacin	1/S	0.25/S	0.25/S
Gentamicin	0.5/S	0.25/S	0.25/S
Tobramycin	8/I	0.25/S	0.25/S
Tigecycline	1/S	0.25/S	0.125/S
Nitrofurantoin	16/S	16/S	16/S
Trimethoprim/sulfamethoxoazole	≥320/R	20/S	20/S

*^*a*^MIC for antimicrobial drugs tested, mg/L. The MICs were interpreted according to the CLSI guidelines.*

### Complete Sequence of pKPC-1713 Plasmid

**FIGURE 3:**
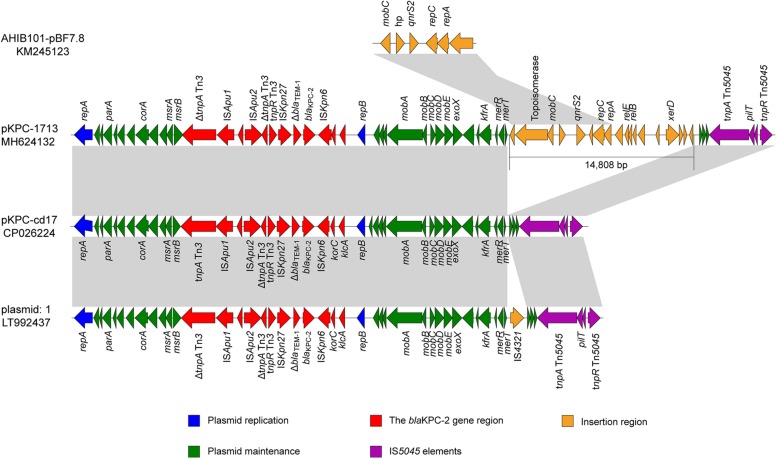
Genomic comparison of p1713-KPC sequence with plasmid pKPC-cd17 (CP026224) from *Aeromonas* sp. ASNIH3, plasmid 1 (LT992437) from *C. Freundii* and plasmid AHIB101-pBF7.8 (KM245123) from *A. hydrophila*. Open reading frames (ORFs) are indicated by arrows, according to their putative functions. The regions sharing a high degree of sequence similarity are shown in gray color.

Sequence analysis of representative pKPC-1713 plasmid revealed the total size of 53,205 bp with an average G + C content of 58% and 66 open reading frames. The plasmid pKPC-1713 harbored five functional regions of genes, including *bla*_KPC–__2_ encoding region, IS*5045* elements, an insertion region, genes involved in plasmid maintenance, and plasmid replication (Figure 3). The search against nr/nt database revealed a 99% identity with *Aeromonas* sp. ASNIH3 IncP-6-type plasmid pKPC-cd17 at 74% coverage, and a 99% identity with *Citrobacter freundii* IncP-6-type plasmid: 1 at 74% coverage (Figure 3). In contrast to pKPC-cd17, an insertion of 14,808 bp DNA fragment and IS*4321* gene were observed between *merT* gene and hypothetical protein in p1713-KPC and plasmid: 1, respectively. The insertion sequence of p1713-KPC harbored 18 different genes, which include the quinolone resistance *qnrS2* gene. Interestingly, BLAST search of this insertion sequence revealed a 99% identity with *A. hydrophila* IB101 plasmid AHIB101-pBF7.8 at 100% coverage (Figure 3). We speculate that p1713-KPC has been formed by an IncP-6 plasmid fusing with the *qnrS* plasmid after one of them has acquired extra DNA with *xerD* etc. Furthermore, *bla*_KPC–__2_ gene was found to be located on a 14.5 kb Tn*3* transposon element in p1713-KPC with the linear structure: Δ*tnpA* Tn*3*-IS*Apu1-*IS*Apu2-*Δ*tnpA*-Tn*3-tnpR* Tn*3-*IS*Kpn27-*Δ*bla*_TEM–__1_*-bla*_KPC–__2_-ΔISK*pn6* (Figure 3). Previous studies of *bla*_KPC–__2_ have indicated its association with Tn*3*-based transposon and responsible for its widespread among *Enterobacteriaceae* in different geographical locations of China (Shen et al., 2009; Yang et al., 2013).

### Plasmid Mobilization

IncP-6 plasmids are found in various species from both clinical and environmental sources, suggesting that they have a broad host range and potential for long-term persistence in the environment (Naas et al., 2013; Dai et al., 2016; Wang et al., 2017; Yao et al., 2017). Interestingly, most of these *bla*_KPC–__2_ bearing IncP-6 plasmids were detected in China. Our findings further revealed that *bla*_KPC–__2_ carrying IncP-6 plasmid has moved into *Aeromonas* spp. Of note, p1713-KPC is lacking of a *tra* module encoding primary pilus, which explains the failure of plasmid conjugation. Studies had shown that plasmids could be able to transfer by conjugation if the right self-transmissible plasmids are present (Smillie et al., 2010). However, due to unknown reasons, mobilization transfer from donor *A. taiwanensis*, which harbored resident plasmid and electrotransformed plasmid containing a *tra* module, to the recipient cells was not successful. Nevertheless, acquisition of free DNA is a general feature of *Aeromonas* isolates (Huddleston et al., 2013). And the IncP plasmid, which is a broad-host-range incompatibility plasmid, has also been proved with the potential to mediate the dissemination of antibiotic resistant genes from the *Enterobacteriaceae* to other Gram-negative bacteria, such as *Pseudomonas aeruginosa* (Zhao et al., 2017). Since the complex mechanism of plasmid transfer is still not fully understood and these 3 IncP plasmids do encode mobilization functions, potential detection of such incP plasmids from clinical isolates should be closely monitored. Therefore, it is suggested that IncP-6 plasmids may act as an important vector responsible for the genetic transmission of *bla*_KPC_ and *qnrS2* among the aquatic *Aeromonas* spp.

## Conclusion

The analysis of plasmids carrying different resistance genes is pivotal for characterization of bacterial isolates, since they play a significant role in transmission of antibiotic resistance (Galata et al., 2018). In this study, we reported the complete sequence of p1713-KPC, a novel IncP-6 plasmid identified from environmental *A. taiwanensis* isolates. To our knowledge, this is the first report describing the co-existence of *bla*_KPC–__2_ and *qnrS2* on the same plasmid in *A. taiwanensis* isolates. The emergence and dissemination of such plasmids in environmental isolates deserve special attention. Given that *Aeromonas* spp. are ubiquitous organisms isolated from a wide range of environmental niches, they might act as important vectors for the dissemination of plasmid-mediated carbapenem- and quinolone-resistance genes. Therefore, potential detection of such plasmids from clinical isolates should be closely monitored.

## Data Availability

The whole genome shotgun project of the *A. taiwanensis* isolates has been deposited into DDBJ/EMBL/GenBank under the Bioproject accession number PRJNA478520. The complete nucleotide sequence of plasmid p1713-KPC has been deposited into the GenBank database under GenBank accession number MH624132.

## Author Contributions

XH, YM, and BZ conceived and designed the experiments. XH, XY, YS, HX, YL, YK and LS performed the experiments. LG, JS, and FY analyzed the data. BZ and XH wrote the manuscript.

## Conflict of Interest Statement

The authors declare that the research was conducted in the absence of any commercial or financial relationships that could be construed as a potential conflict of interest.

## Footnotes

1 https://cge.cbs.dtu.dk/services/

## Supplementary Material

FIGURE S1 PFGE profiles of *A. taiwanensis* strains. A dendrogram of PFGE profiles was constructed with BioNumerics v7.6 by using UPGMA (unweighted pair group method with averages) clustering. Isolates with a similarity cut-off of ≥80% were considered as pulsotypes. The scale bar indicates the percentage of genetic relatedness.

TABLE S1 Primers used in the plasmid sequencing of novel IncP-6 plasmid pKPC-1713, harbouring *bla*_KPC–2_ and *qnrS2* genes.
