# Supplementary figures and images for: Emergence and Characterization of a Novel IncP-6 Plasmid Harboring blaKPC–2 and qnrS2 Genes in Aeromonas taiwanensis Isolates
Source: Front Microbiol. 2019 Sep 12;10:2132. doi: 10.3389/fmicb.2019.02132 (PMC6751286; doi:10.3389/fmicb.2019.02132)

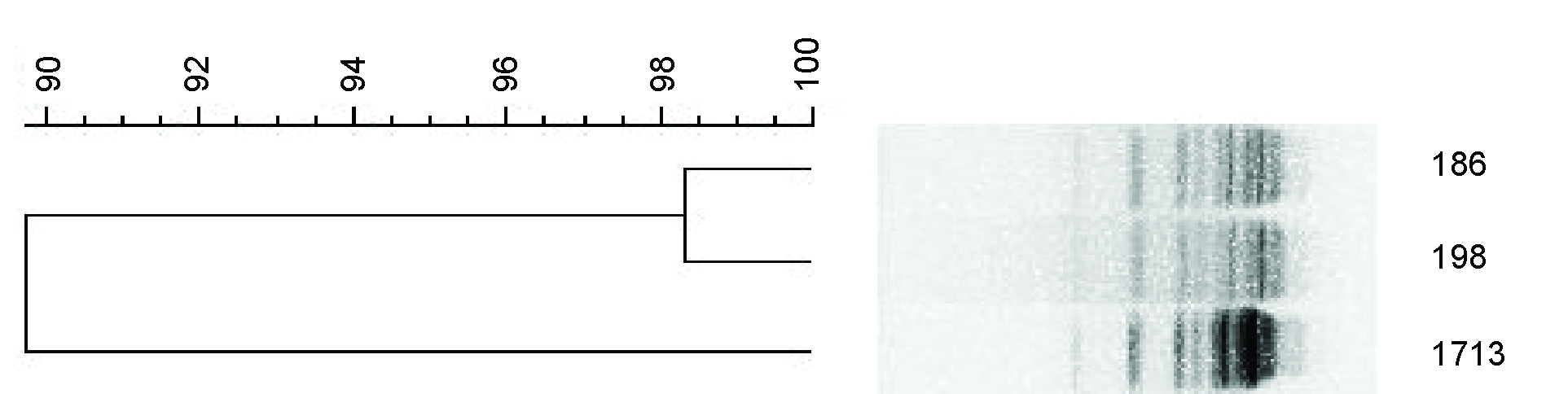

Supplement: FIGURE S1 — PFGE profiles of A. taiwanensis strains. A dendrogram of PFGE profiles was constructed with BioNumerics v7.6 by using UPGMA (unweighted pair group method with averages) clustering. Isolates with a similarity cut-off of ≥80% were considered as pulsotypes. The scale bar indicates the percentage of genetic relatedness. [file Image_1.TIF]
