# Supplementary material for: Emergence and Characterization of a Novel IncP-6 Plasmid Harboring blaKPC–2 and qnrS2 Genes in Aeromonas taiwanensis Isolates
Source: Front Microbiol. 2019 Sep 12;10:2132. doi: 10.3389/fmicb.2019.02132 (PMC6751286; doi:10.3389/fmicb.2019.02132)
Supplement: TABLE S1 — Primers used in the plasmid sequencing of novel IncP-6 plasmid pKPC-1713, harbouring blaKPC–2 and qnrS2 genes. [file Table_1.DOCX]

Supplementary Material

# Supplementary Data

# Table S1. Primers used in the plasmid sequencing of novel IncP-6 plasmid pKPC-1713, harbouring *bla*_KPC-2_ and *qnrS2* genes

| **Primer ID** | **Sequences** | **Primer pairs** |  |
| --- | --- | --- | --- |
| **Primers for plasmid sequencing** | | | |
| p1713-1154F | TCGTCGTAGCAGCGGAACA | A |  |
| p1713-2619R | GCAACCTCTGACCTCGTGGA | A |  |
| p1713-2384F | TCGCTCGTCGGCTCTTGGT | B |  |
| p1713-3814R | GGTAGGTCCCTCGTTCGGTA | B |  |
| p1713-11219F | TCTCGCCCGTCACAAACC | C |  |
| p1713-12731R | CAACCTCAGTCAGGAGAACCC | C |  |
| p1713-27207F | ACGCTTGACCAGATTTCCG | D |  |
| p1713-29939R | GCGACAGCGATTCACAAAA | D |  |
| p1713-39662F | GCAATCAGGGGATTCAAGGC | E |  |
| p1713-41144R | TACATCGCGATTGGTGGACA | E |  |
| p1713-3491F | TCGACGACTGCTCCGTGAAG | F |  |
| p1713-4874R | TGGCTACTTCGTCGTGCTC | F |  |
| p1713-4654F | TGAATGACTGGACTGCTTCTAGA | G |  |
| p1713-6159R | GGGCAGCTCATCTCAACAAC | G |  |
| p1713-5978F | CCGAGAAGACGCCTGGATT | H |  |
| p1713-7487R | GGGCGAGTACAACGACAACAAG | H |  |
| p1713-7171F | ATCCCTCGGACTCCATCTCG | I |  |
| p1713-8713R | GCATCAACAAAACGCTGTATCTG | I |  |
| p1713-8508F | CACGACGGCGTTAGTGACC | K |  |
| p1713-11567R | AAGCCTGAACCCCAAGATAA | K |  |
| p1713-12469F | AGGGTTAGGGTGAGGGGAGC | L |  |
| p1713-15191R | GTCGGTACTGATCCCGTCAT | L |  |
| p1713-14901F | TTTGGTTACGCTCGGGTCT | M |  |
| p1713-17786R | AGATGGGTGACCACGGAAC | M |  |
| p1713-17563F | CGCTAAACTCGAACAGGACTT | N |  |
| p1713-20352R | ATTACCGCTTGCCTCTACCTCTA | N |  |
| p1713-20109F | CCCCTGTGATTGGTTGCAG | O |  |
| p1713-22894R | ACGGCCTTGCAGACCCTAA | O |  |
| p1713-22641F | ACAACAGCCTTCAAACCGC | P |  |
| p1713-25309R | GCCCTATCCCGACCTGGAG | P |  |
| p1713-24969F | CCTTGTATTCGTCGTTCCCG | Q |  |
| p1713-27409R | GGTGAATGTCGCCTTTGATGTAT | Q |  |
| p1713-29730F | TCGTACTCTCCATGCCACTCG | R |  |
| p1713-32666R | CTAGGTGCTATTCTAAACTCCGTA | R |  |
| p1713-32460F | ACGCAGTCGCTTCACCATG | S |  |
| p1713-35160R | CAGAAATCAGGGGTGACTTATG | S |  |
| p1713-34954F | GTCACGTCCTGTTGGAGTCG | T |  |
| p1713-37902R | AGGAACCCAATAGATTACTGATG | T |  |
| p1713-37642F | GCTCAATGGCTACGTTATCCG | U |  |
| p1713-39884R | AGGATGCGATGGTGGTCAAG | U |  |
| p1713-40716F | TCAAGGGTCAGGTTCTCGG | V |  |
| p1713-43428R | CGCAGTTTTGAGATCCTTGATACT | V |  |
| p1713-43206F | CCCCACAAATAATCCATAACAC | W |  |
| p1713-46027R | GCGAGCCCAATAACGGTG | W |  |
| p1713-45830F | TACCTGGGATAAGGAGGACAC | X |  |
| p1713-48786R | CCTACTTGGCTACCACTTCAGTC | X |  |
| p1713-48552F | GGTGGGTCTATCTCCCGTTT | Y |  |
| p1713-51520R | TGCGGGTCGTGTTGGATAC | Y |  |
| p1713-51300F | CACAGCCCGTTGCAGGTTA | Z |  |
| p1713-239R | GCACAGATGGGCATTAGCA | Z |  |
| p1713-52950F | CCAGGTTCACGTTTCCAGTACA | AA |  |
| p1713-1407R | CAAGAAGCAGGACAGTTACCG | AA |  |
| **Primers for confirmation of plasmid sequence** | | | |
| p1713-20109-W2F | ATCTAAACGGCTTTACACCT |  |  |
| p1713-43206-W2R | TACGTTTTGATGCCCCATCC |  |  |
| W1F-0823-P099-C01 | GGTACGCAATGGACACTTCG |  |  |
| W1R-0823-P099-D01 | GCCTTGCACGGTTTTGTTCAG |  |  |
| W1F-p1713-17563 | AAGGATGACAAGCACAGCGAG |  |  |
| W1R-p1713-17563 | GCTTATGGTGCCTGGTCTATCT |  |  |
| W1F-p1713-22641 | CTTCTTGGTGTGGGGCAATTG |  |  |
| W1R-p1713-22641 | GACAAGAAGCATCACATCCTCATC |  |  |
| W1F-p1713-24969 | CGAGTCGCTTTTGTAACTTGGAT |  |  |
| W1R-p1713-24969 | AGAAGGCCCGCTTGAAAGAG |  |  |
| W1R-p1713-8508 | TGGATAAGCAACGAACGTCCT |  |  |
| W1F-p1713-8508 | GATTGTTCTGACTGGCGTAACA |  |  |
| W1R-0823-P099-E11 | GAATCATATTCCTTCCGGCG |  |  |
| W2R-0823-P099-F11 | TATACTTTCCCGAACTCACA |  |  |
| W3R-0823-P099-G11 | GTAGTCTCGCAGGCTGGATA |  |  |
| W1R-0823-P099-H11 | CCCAGTGCTGCAATGATACC |  |  |
| W2R-0823-P099-A12 | ACGGGTGTTTTGTGTTGGAG |  |  |
| W3R-0823-P099-B12 | CACCACGATGAGCTCCTG |  |  |
| p1713-20109-W1F | GGGCGCGGTAGTTGATCC |  |  |
| p1713-52950-W1R | GTGGGATCGTCTGGAGGA |  |  |
| p1713-51300-W1F | CTACAAGTCGATGCCCTGAAAC |  |  |
| p1713-48552-W1F | GCGGATCAAGGGCTCCAG |  |  |
| p1713-48552-W1R | ATCATCGCCGCTGGATAC |  |  |
| p1713-45830-W1F | GCCCACCTCGCCAAGATC |  |  |
| p1713-45830-W1R | TGATCCCCCTAAACGCTATGAG |  |  |
| p1713-43206-W1F | GAATTTCCCCAAACACAGCCAG |  |  |
| p1713-43206-W1R | ATTGGGCAGGGAACCTG |  |  |
| p1713-40716-W1F | TCAAACACAATGCAGGAGACATG |  |  |
| p1713-40716-W1R | AGGGGGCGTTACGTTAAATCG |  |  |
| p1713-29730-W1R | CCTGTTCAGATTGTCATTTGTGGA |  |  |
| W1F-p1713-32460 | ATCTTCACCGGCCAGATAGAC |  |  |
| W1R-p1713-32460 | GAACTAGATCCGACCGCCTAC |  |  |
| W1F-p1713-37642 | GCAAACTTTAGTAACGCCAATTG |  |  |
| W1R-p1713-37642 | GGAGGCATCTTTTCAACTCACTG |  |  |
| p1713-29730-W1F | CAGAATGACACTTTACCCGC |  |  |
| p1713-34954-W1F | GCGTATTGGTCTGGTCAA |  |  |
| p1713-34954-W2F | CTTGGATTCGTCAGTGTTCA |  |  |
| p1713-34954-W3F | GCCGAGTGTATTACGCTAAG |  |  |
| p1713-34954-W1F | CATGAAGTCAGTCGTCACGG |  |  |
| p1713-34954-W2F | CGCTTGGATTCGTCAGTGTT |  |  |
